# Is female preference for large sexual ornaments due to a bias to escape predation risk?

**DOI:** 10.1186/1471-2148-12-33

**Published:** 2012-03-13

**Authors:** Zhen Zhu, Tae Won Kim, Jae Chun Choe

**Affiliations:** 1 Laboratory of Behavior and Ecology, Division of EcoSicence, Ewha Womans University, Seoul 120-750, South Korea; 2 The Jane Goodall Institute China Office/Roots & Shoots Beijing Office, (Located inside Beijing City International School, Room 1309), No. 77, Bai Zi Wan Nan 2 Rd, Chao Yang District, Beijing, P. R. China; 3 Monterey Bay Aquarium Research Institute, 7700 Sandholdt Road, Moss Landing CA95039, USA

## Abstract

**Background:**

A female preference for intense sexual visual signals is widespread in animals. Although the preferences for a signal *per se *and for the intensity of the signal were often regarded to have the identical origin, no study has demonstrated if this is true. It was suggested that the female fiddler crabs prefer males with courtship structures because of direct benefit to escape predation. Here we tested if female preference for both components (i.e. presence and size) of the courtship structure in *Uca lactea *is from the sensory bias to escape predation. If both components have the identical origin, females should show the same response to different-sized courtship structures regardless of predation risk.

**Results:**

First, we observed responses of mate-searching female *U. lactea *to courting males with full-sized, half-sized and no semidomes which were experimentally manipulated. Females had a directional preference for males with bigger semidomes within normal variation. Thereafter, we tested the effect of predation risk on the female bias in the non-courtship context. When threatened by an avian mock predator, females preferentially approached burrows with full-sized semidomes regardless of reproductive cycles (i.e. reproductive periods and non-reproductive periods). When the predator cue was absent, however, females preferred burrows with semidomes without discriminating structure size during reproductive periods but did not show any bias during non-reproductive periods.

**Conclusions:**

Results indicate that selection for the size of courtship structures in *U. lactea *may have an origin in the function to reduce predation risk, but that the preference for males with structures may have evolved by female choice, independent of predation pressure.

## Background

Sexual selection can be used to explain the evolution of various male secondary sexual characters which are used as courtship signals [1,2]. A number of experimental studies provide evidence that females prefer males with conspicuous sexual traits [3-9]. Females may benefit from choosing males with strong signals directly by reducing their own predation risk [10-14] or by providing better parental care to their offspring [15-17]. Females also may acquire indirect benefit by transmitting high genotypic quality of males to their offspring [3,6,18,19]. The preference for the existence of the signal and the preference for signal intensity are often regarded to have the same origin [8,20-23]. However, the preferences for a signal *per se *and for intensity of the signal may have different origins when the two components serve different functions. No studies to date have tried to separate the origins of preferences for these two components of courtship signals.

Fiddler crabs of the genus *Uca *are semi-terrestrial animals that live on intertidal mud or sand flats [24]. Courting males of approximately 18 species sometimes build various kinds of mud or sand structures at their burrows such as hoods, pillars, and lips [25]. It has been demonstrated that the mud pillars in *U. beebei *[26-28] and the sand hoods in *U. musica *[25,29] function as sexual signals in attracting females. The sensory trap hypothesis has been suggested to explain the female preference for particular courtship structures [12,30]. It was recently found that the preference for hoods in *U. terpsichores *[11] and for pillars in *U. beebei *[10] could increase with the perceived predation risk, because the structures provided a direct survival benefit to females by allowing them to escape predation. However, female *U. terpsichores *did not show a directional preference for exaggerated artificial hoods which were conspicuously larger than average-sized natural hoods [30]. Nevertheless, females may prefer larger structures within natural variation and this possibility has not yet been explored.

Here we tested if both the preference for a courtship structure and the preference for the size of the structure in the fiddler crab *Uca lactea *were shaped by the need of females to escape predation. Males of *U. lactea *build semidomes using mud at their burrows and wave their large claws to attract females for mating. Previous studies suggest that semidome building in *U. lactea *is related to courtship signaling [31] and the semidome has a function to attract females [32]. Given the evidence of previous studies, females may prefer larger semidomes over smaller ones if the courtship structure could provide a greater survival benefit to them.

Our first objective in this study is to determine if female *U. lactea *prefer males with larger sized courtship structures. If male attractiveness increases with the semidome size, we can predict that males with larger semidomes would mate more successfully than those with smaller semidomes. The second objective of this study is to examine if the preference for structures and preference for structure size have the same origin to function in predation avoidance [28,29]. To test this, we conducted an arena experiment in the non-courtship context for females to choose burrows with different-sized semidomes in the presence and absence of predation cue. A previous pilot study found that female *U. lactea *without predator threat preferentially moved to burrows with semidomes during reproductive periods, but did not show any orientation bias during non-reproductive periods [32]. Thus, we conducted an experiment with and without a predator cue during both reproductive and non-reproductive periods to determine if the reproductive cycle influences female responses to male courtship structures.

## Results

### Female preference in the context of mate choice

We observed 159 interactions between 73 females and semidome-building males with full-sized semidomes, half-sized semidomes and without semidomes (Table 1). On average each female visited 1.68 ± 1.32 males and passed 0.49 ± 0.88 males before choosing one of them as a potential mate.

**Table 1:** Responses of mate-searching females to courting male *U. lactea *with full-sized, half-sized or without semidome

Type of semidome	Visiting	Passing	Totals	% visiting
full-sized	61	6	67	91.04
half-sized	37	12	49	75.51
without semidome	25	18	43	58.14

Generalized linear mixed model (GLMM) analyses revealed a significant difference in attractiveness among semidomes of different size (effect of semidome size: *F*_2, 156 _= 6.879, *p *= 0.001, effect of female identity as a random factor: *Z *= 0.654, *p *= 0.513). The visiting frequency of mate-searching females showed a linearly increasing trend with semidome size (*X*^2 ^= 16.313, d.f. = 1, *p *< 0.001) in the chi-square test. Females preferred males with semidomes of any size to males without semidomes (*X*^2 ^= 19.008, d.f. = 1, *p *< 0.001). Also, males with full-sized semidomes were significantly more attractive than males with half-sized semidomes (*X*^2 ^= 5.210, d.f. = 1, *p *= 0.022).

### The effect of predation risk on female choice for different sized-semidomes

An ordinal logistic regression test revealed that predation risk is a significant factor which influences the female choice on semidome size (Wald *X*^2 ^= 6.606, d.f. = 1, *p *= 0.010). However, there was no marked effect of reproductive cycle (i.e., reproductive or non-reproductive period) on female choice (Wald *X*^2 ^= 0.001, d.f. = 1, *p *= 0.975) in the ordinal regression test. The effect of interaction between predation pressure and reproductive cycle was not significant either (Wald *X*^2 ^= 2.721, d.f. = 1, *p *= 0.099).

**Figure 1:**
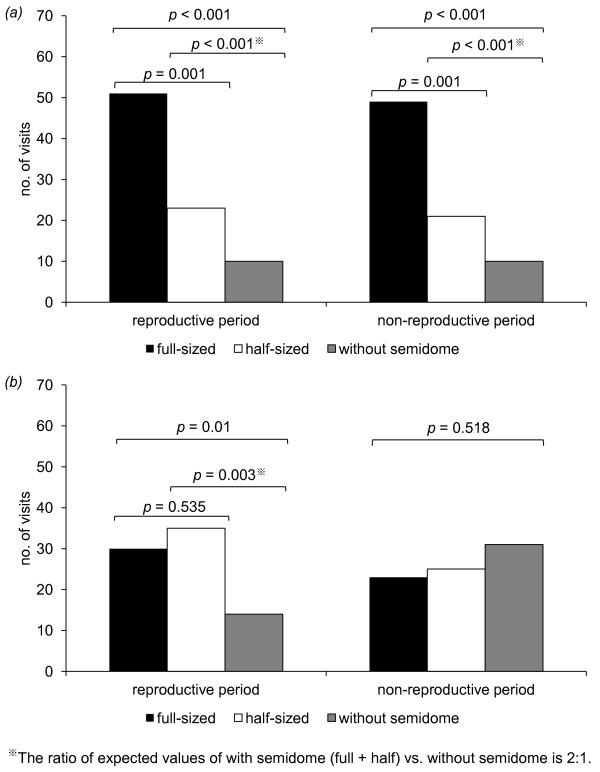
**Visiting frequency by females to artificial burrows with full-sized, half-sized or without semidome under *(a) *predator-present condition and *(b) *predator-absent condition in the arena test**.

Under high predation risk, female choices did not differ significantly between reproductive periods and non-reproductive periods (*X*^2 ^= 0.033, d.f. = 2, *p *= 0.983). Under low predation risk, however, there was significant difference between two periods (*X*^2 ^= 9.013, d.f. = 2, *p *= 0.011). More females moved to burrows with full-sized semidomes than to burrows with half-sized semidomes or without semdiomes in the presence of a mock predator (*X*^2 ^= 61.659, d.f. = 2, *p *< 0.001, Figure 1a). In the absence of a mock predator, however, females showed different orientation biases varying with the reproductive cycle (Figure 1b): females during reproductive periods preferred to move to burrows with semidomes rather than to burrows without semidomes (*X*^2 ^= 8.665, d.f. = 1, *p *= 0.003), but did not discriminate between the sizes of semidomes (*X*^2 ^= 0.385, d.f. = 1, *p *= 0.535); during non-reproductive periods, females did not show any orientation bias to different semidome treatments (*X*^2 ^= 1.316, d.f. = 2, *p *= 0.518).

## Discussion

Our study provides the experimental evidence that sexual attractiveness of male fiddler crabs increases with size of the courtship structures within natural variation. Compared to males with half-sized semidomes and without semidomes, those with full-sized semidomes were more attractive to mate-searching females. Males with half-sized semidomes were more attractive to females than those without semidomes. The directional preference for the size of structures suggests that there is a positive correlation between male attractiveness and semidome size within the normal range of variation.

Why do females prefer males with larger structures? Given that females in the non-courtship context showed a directional orientation bias for larger semidomes under predation threat regardless of the reproductive cycle, larger semidomes may be more salient visual cues to provide protection to females. The consistency between female choice in the mate-searching context and female orientation response in the predation context [12,28,29] indicates that the preference for the larger signal may have originated from the female bias to escape predation. In other words, mate choice is linked to male-built structures that can serve as a landmark to keep females safe against predators. Characteristics of the fiddler crab's visual system may also explain why females prefer larger semidomes. The horizontal resolving power of fiddler crabs' eyes is poor compared to their vertical resolving power [33-35]. Accordingly, fiddler crabs are better able to resolve objects in the vertical than in the horizontal plain. Semidomes which are tall enough to be imaged within the zone of the acute vertical resolution of their eyes may be most conspicuous to fiddler crab females [36]. In the absence of predator cues, however, females responded differently to different-sized semidomes depending on the reproductive cycle. During the non-reproductive periods, females did not show any difference in the preference for burrows with different treatments. In contrast, during the reproductive periods, they showed different responses. Although females did not more preferentially move to full-sized structures than half-sized structures, they did prefer moving to burrows with structures over moving to burrows without structures. This result presents a striking contrast to the case of *U. musica *(later correctly identified as *U. terpichores*) where females have an orientation preference to structures, even without predator cue [29]. Therefore, the predator-induced sensory trap hypothesis [12] cannot fully explain the female preference on the structure building in *U. lactea*, as the effect of large structures decreased in the absence of a predator. Rather, structure building itself may have been selected by female choice independent of predation pressure.

This female preference for structures may have started because fiddler crab courtship structures may signal male nutritional condition. In previous studies in which males were supplemented with food, it was shown that structure building depends on male condition [37-39]. The semidome building behavior may signal phenotypic condition of a male crab, which could be affected by food availability. Females may choose better males using semidomes as an indicator of genetic quality. Later, the predation pressure might enhance the selection on larger structures, which may help reduce predation risk for female crabs.

## Conclusions

In conclusion, we have decoupled the origins of the preference on the sexual signal and signal intensity by manipulating predation risk. The preference for semidomes in *U. lactea *may have been selected by female choice not related with predation pressure. However, the preference for increased semidome size could have been strengthened to reduce the predation risk for females. Our results suggest that sexual signal *per se *and signal intensity is not necessarily selected by the same selection pressure.

## Methods

### Study site and species

All observations and experiments were conducted at the 'Choji' intertidal mudflat (37°35'N 126°32'E) in Ganghwa Island near the Han River in South Korea from June to August in 2007-2009. *U. lactea *lives on mudflats of the upper intertidal zone in the temperate, tropical and subtropical Indo-Pacific [24]. From May to October, they emerge and feed on the mudflat surface when the tide recedes. Each reproductive cycle of about 15 days has reproductive and non-reproductive periods in the study site [40]. During the breeding season, males build semidomes on the mudflat at their burrows, and then wave their large claws in order to attract females to mate. When the females are ready to mate, they leave their burrows and wander on the mudflat, searching for mates. Receptive females approach courting males, enter the burrows following them, and then leave the burrows several seconds later. A female visits and samples several burrows of courting males until she eventually selects a male as her mate. In successful courtship, the male copulates with a female underground and plugs the entrance of his burrow with mud for mate guarding. The mating pair remained in the burrow together for one to five days [41]. After the female finished ovulation, the male comes out from the burrow and departs, leaving the female in the male's burrow to incubate her eggs. In the non-reproductive periods, both males and females mostly feed on the mudflats, males do not wave claws to attract females and females do not search for their mates [40].

Semidome building behavior of the males starts at the beginning of the breeding season in early June. During 3-5 day periods when the number of courting males peaks in each semi-lunar cycle, many males construct semidomes using mud at their burrows. The mean ± SD height and width of semidomes was 17.6 ± 5.0 mm and 26.7 ± 5.6 mm [31].

The eastern Mew Gull, *Larus canus*, is the natural predator of *U. lactea *at the study site. During the whole experiment period they were rarely present at test areas.

### Female preference test in the context of mate choice

We tested the effect of courtship structures on the female preference by measuring the visiting and passing frequencies of female crabs. We defined visiting frequency as the number of times mate-searching females approached and touched a male burrow with a part of their bodies. The passing frequency was defined as the number of times mate-searching females walked towards a male burrow but turned away before getting close to it and left without a contact with the burrow. As the mating rates of males might be positively correlated with the visiting rates of mate-searching females [10,42], by recording the visiting frequency, we are able to give reliable estimates of attractiveness of males with different sized courtship structures. We compared female choice among males with full-sized semidomes, half-sized semidomes and without semidomes.

The experiment was conducted during the intense courtship period in which most of males constructed semidomes at their burrows. We selected an observation plot (approx. area: 150 m^2^) randomly in each experimental day. For every three adjacent actively courting males with semidomes in the plot, we removed all the semidomes already built and replaced them with full-sized, half-sized artificial semidomes, and nothing respectively. We deployed 50-70 manipulated clusters of courting males in randomly chosen plots. Thus, different-sized semidomes were randomly distributed across the observation area. We made the artificial semidomes prior to the experiment. Artificial semidomes were made out of clay to imitate natural semidomes, and colored with khaki dyestuff outside. We produced artificial semidomes in two different sizes: full-sized semidomes (17 mm/25 mm in height/width) and half-sized semidomes (8.5 mm/25 mm in height/width). In order to easily identify the three different experimental groups of males (with full-sized, half-sized, and no semidomes) from a distance, we marked each burrow with one of three colored (red, yellow and blue) wooden sticks (diameter: 2 mm; length: 10 cm) by inserting a wooden stick 10 cm away from each burrow. We daily designated and switched the color for each experimental group in a random order to control the effect of color of wooden sticks on female choice.

We sat on a chair near the plot and observed crabs directly or through binoculars. The distance between the observer and test plot was kept to at least 1.5 m so that the crabs could behave normally and would not be affected by our observation. We recorded the number of times females visited males with the different-sized semidomes and also noted whether females left or stayed in the male burrow. The observation on each mate-searching female continued until she left the experimental area or entered a burrow and did not emerge within five minutes. A total of 159 responses from 73 females were recorded. We did not identify individual females, but the probability that our tally included some repeated records by the same females should be small. While searching for a mate, females seldom visit a given male twice. Therefore each visit can be considered as a unique courtship interaction of a given male-female pair. This assumption has been confirmed in previous studies [10,11,25]. Since the original semidomes built by males were removed and replaced by different-sized artificial ones randomly in this experiment, we supposed that there was no difference in the male behavior and activity of each male-female courtship interaction between males with different semidome size.

### Female choice for different-sized semidomes in the non-courtship context

We selected an area uninhabited by crabs to avoid the influence of interactions with other crabs, and scratched a 50 cm-diameter circle onto the mudflat. We made 12 artificial burrows on the edge of the circle at even intervals. The artificial burrows were approximately 10 mm in diameter and 15 mm in depth, which were large enough to let females in. Full-sized and half-sized semidomes were placed behind the artificial burrows relative to the arena center. The distance from the base of a semidome to the burrow edge was approximately 5 mm. For every three adjacent burrows, one was ornamented with a full-sized semidome, another had a half-sized semidome and another had no semidome. The order of semidome placement was different in every set of three adjacent burrows.

We caught females by placing a long plastic stick over the burrow entrance after the female emerged from the burrow and then catching the crab by hand. For each test procedure, a female was placed in the center of the arena. The female was randomly either exposed to a mock predator (the predator-present condition) or not (the predator-absent condition). We used a bird-shaped model (approx. volume: 1 dm^3^) connected to the end of a fishing rod by 1 m-long nylon thread to imitate a natural predator. When we flung the fishing rod over the experimental set, the mock predator moved in random directions. We waited until the crab either approached a burrow or left the arena. We recorded which of three kinds of burrows (with full-sized, half-sized, or no semidomes) females visited or touched. Each female was used once. The female which did not select the burrow was not counted. A total of 163 females during reproductive periods and 159 females during non-reproductive periods moved to burrows in this study.

### Statistical analysis

All analyses were conducted using IBM SPSS Statistics 20. To determine whether there was a significant difference in the frequency of female visits among males with full-sized semidomes, half-sized semidomes and without semidomes, we ran a generalized linear mixed model (GLMM) with female choice (coded "visiting" as 1, and "passing" as 0) as a dependent variable, semidome size as a fixed effect, and female identity as a random factor. We additionally used a chi-square test for trend to evaluate if a linear trend exists between semidome size and visiting frequency. Given that the size of artificial semidomes is ordinal in the arena test, the categories of female responses to semidomes can be ordered in a meaningful way 'full-sized/half-sized/without semidome'. Therefore we used ordinal logistic regression [43,44] to analyze relationships both between female choice and predation risk as well as between female choice and reproductive cycle. Then, we used a chi-square test to compare female choices between reproductive periods and non-reproductive periods under each predation risk. If female responses were significantly different between two periods, we tested the effect of semidome size on female choice separately for each period. If responses were not different, we pooled data to test the effect of semidome size on female choice.

#### Ethical note

The Institutional Animal Care and Use Committee, which oversees animal experimentation at Ewha Womans University, was not established when this study was designed and conducted. However, all experimental manipulations and procedures complied with recommended guidelines for the treatment of animals in behavioural research [45]. We did not sacrifice any animals for this study. In the end of each test, female crabs were released in the same areas where they captured and all of them behaved normally.

## Authors' contributions

ZZ designed the study, did field work, analyzed data and wrote the paper. TWK participated in the design of the study, did field work and helped to draft the manuscript. JCC participated in the design of the study and writing of the manuscript. All authors approved the final version of the manuscript.
